# Age-Associated Glia Remodeling and Mitochondrial Dysfunction in Neurodegeneration: Antioxidant Supplementation as a Possible Intervention

**DOI:** 10.3390/nu14122406

**Published:** 2022-06-09

**Authors:** Anna Picca, Evelyn Ferri, Riccardo Calvani, Hélio J. Coelho-Júnior, Emanuele Marzetti, Beatrice Arosio

**Affiliations:** 1 Fondazione Policlinico Universitario “Agostino Gemelli” IRCCS, 00168 Rome, Italy; anna.picca@policlinicogemelli.it (A.P.); riccardo.calvani@guest.policlinicogemelli.it (R.C.); emanuele.marzetti@policlinicogemelli.it (E.M.); 2 Fondazione IRCCS Ca’ Granda Ospedale Maggiore Policlinico, 20122 Milan, Italy; 3 Department of Geriatrics and Orthopedics, Università Cattolica del Sacro Cuore, 00168 Rome, Italy; coelhojunior@hotmail.com.br; 4 Department of Clinical Sciences and Community Health, University of Milan, 20122 Milan, Italy; beatrice.arosio@unimi.it

**Keywords:** astrocytes, extracellular vesicles, inflammation, microglia, mitochondrial quality, mitophagy, polyphenols, neurodegenerative diseases, oxidative stress, vitamins

## Abstract

Aging induces substantial remodeling of glia, including density, morphology, cytokine expression, and phagocytic capacity. Alterations of glial cells, such as hypertrophy of lysosomes, endosomes and peroxisomes, and the progressive accumulation of lipofuscin, lipid droplets, and other debris have also been reported. These abnormalities have been associated with significant declines of microglial processes and reduced ability to survey the surrounding tissue, maintain synapses, and recover from injury. Similarly, aged astrocytes show reduced capacity to support metabolite transportation to neurons. In the setting of reduced glial activity, stressors and/or injury signals can trigger a coordinated action of microglia and astrocytes that may amplify neuroinflammation and contribute to the release of neurotoxic factors. Oxidative stress and proteotoxic aggregates may burst astrocyte-mediated secretion of pro-inflammatory cytokines, thus activating microglia, favoring microgliosis, and ultimately making the brain more susceptible to injury and/or neurodegeneration. Here, we discuss the contribution of microglia and astrocyte oxidative stress to neuroinflammation and neurodegeneration, highlight the pathways that may help gain insights into their molecular mechanisms, and describe the benefits of antioxidant supplementation-based strategies.

## 1. Introduction

The central nervous system (CNS) is composed of a heterogeneous population of cells that hold unique features and cooperate with neurons for proper CNS function. Neurons are highly specialized cells in charge of transmitting, processing, and storing information and are indicated as “functional cells of the brain”. Non-neuronal cells, including microglia and macroglia (i.e., astrocytes, ependymal cells, and oligodendrocytes), perform other vital functions within the CNS [1].

Owing to its hematopoietic origin, microglia is the primary immune source in the CNS that helps nourish and support neurons by clearing neuronal debris and responding to environmental stimuli [2,3]. Upon infection, trauma, or neurodegeneration, microglial cells become activated, undergo rapid reshaping, including changes in gene expression and function, and are recruited at the site of injury [4,5]. Here, they proliferate and phagocyte damaged cells and cellular debris [4]. As a result of their activation, microglial cells produce high levels of pro-inflammatory mediators, including cytokines (e.g., tumor necrosis factor α (TNFα), interferon-γ (IFN-γ), interleukin-6 (IL-6)), chemokines (e.g., monocyte chemoattractant protein-1 (MCP-1)) [6], reactive oxygen species (ROS), and nitric oxide species (NOS) [7], with cytotoxic effects in case of prolonged production [3]. Anti-inflammatory cytokines (e.g., IL-10, IL-1Rα, and transforming growth factor beta-β (TGF-β)) down-regulate microglia activation [8,9]. As such, microglia has been attributed major roles in neuronal survival and the modulation of neuroinflammation, ultimately contributing to promoting neuronal homeostasis and limiting the onset and progression of neurodegeneration [10].

Astrocytes, similar to microglial cells, are involved in a wide spectrum of functions, including the provision of metabolic substrates to neurons for adequate synaptic activity, synthesis and recycling of neurotransmitters, diffusion of glutamate-induced excitatory signals [11,12,13], and interaction with endothelial cells of the blood–brain barrier [14]. Indeed, since there is no direct contact between neurons and microvessels, some essential substrates (e.g., glucose and oxygen supplied by the cerebral circulation) are delivered to neurons by astrocytes [15].

Aging induces substantial remodeling of glial cells. In particular, changes in microglia density, morphology, cytokine expression, and phagocytic capacity have been observed [5,16,17]. Furthermore, age-related modifications of glial intracellular composition, including hypertrophy of lysosomes, endosomes and peroxisomes, and the progressive accumulation of lipofuscin, lipid droplets, and other debris have been reported [18,19,20,21]. These phenotypic alterations have been associated with a significant decline of microglial function with a reduced ability to survey the surrounding tissue, impaired synapses activities, and poor recovery from injury [3,22]. Aged astrocytes also show reduced capacity to support metabolite delivery to neurons, thus affecting neuronal viability [23]. Moreover, the concentration of glutamate and glutamate-aspartate transporters, and glutamine synthase are reduced in senescent astrocytes, leading to dysfunctional glutamate regulation [24,25].

In the setting of reduced glial activity, several stressors and/or injury signals can trigger a coordinated action of microglia and astrocytes that may amplify neuroinflammation and contribute to the release of neurotoxic factors [26]. In this regard, an overall decline in processes involved in preserving cell’s quality may play a major role. Indeed, oxidative stress and proteotoxic aggregates, which are cleared in physiological conditions, may burst astrocytes-mediated secretion of pro-inflammatory cytokines (e.g., IL-6) [27], thus activating microglia and favoring microgliosis during aging and associated conditions [28]. In the context of persistent stimuli, sustained microglia activation and inflammation can make the brain more susceptible to injury and/or neurodegeneration. A deeper understanding of the pathophysiological mechanisms underlying this age-associated decline in cell quality during neurodegeneration is highly sought after, as it may support the development of novel therapeutic strategies.

Here, we discuss the contribution of microglia and astrocyte oxidative stress to neuroinflammation and neurodegeneration, highlight the pathways that may help gain insights into their molecular mechanisms, and describe the benefits of antioxidant supplementation strategies.

## 2. Oxidative Stress in Microglia and Astrocytes: The Contribution of Mitochondrial Dysfunction

Age-related mitochondrial dysfunction and declines in processes preserving cell’s quality induce microglia and astrocytes alterations that ultimately compromise their ability to support neuronal health and respond to environmental stimuli. Greater oxidative stress and accumulation of proteotoxic aggregates may burst astrocyte-mediated inflammation [27], thus activating microglia and favoring microgliosis [28].

The integration and regulation of a plethora of signals, including ROS and immune regulation, are pivotal for achieving a cooperation between microglia and astrocytes aimed at preserving brain homeostasis [29]. ROS/RNS, which have long been considered detrimental molecules, are now regarded as relevant signaling factors that modulate CNS activities [30,31]. However, if overproduced, in conditions of cell dysmetabolism, mitochondrial dysfunction, or calcium overload, ROS and RNS can inflict damage to cell’s structures and macromolecules, including lipids, proteins, and DNA [32].

Mitochondria are among the major cellular sources of ROS through the activity of the electron transport chain (ETC) [33]. Plastic and highly interconnected mitochondria occupy the main body of glial cells and the long processes of astrocytes. However, different from neurons, these cells synthesize most of their ATP from glycolysis. The low reliance of astrocytes on oxidative metabolism is partly explained by the organization of their ETCs with a small percentage of complex I-organized supercomplexes [34]. This ETC rearrangement is associated with higher rates of ROS production in astrocytes compared with neuronal ETC [34]. Notwithstanding, well-functioning mitochondria are pivotal in cell’s activities other than metabolic purposes. For instance, mitochondrial biogenesis has been implicated in the regulation of astrocyte maturation and synaptic pruning [35]. Conversely, the deletion of the mitochondrial m-AAA protease in astrocytes of mice has been reported to induce neurodegeneration [36]. On a similar note, dysfunction and fragmentation of mitochondria in microglial cells have also been implicated in neurodegeneration [37]. In particular, the extent of mitochondrial damage and the release of dysfunctional and fragmented organelles have been associated with the ability of triggering neuronal damage and propagating neuronal death via the activation of naïve astrocytes into the pro-inflammatory A1 state [37].

Mitochondrial dysfunction and associated oxidative stress have also been related to the accrual of misfolded proteins in neurodegenerative conditions, among which Parkinson’s disease (PD) is actively investigated [38]. In particular, α-synuclein (α-syn) aggregation has been indicated as a relevant mechanism in both familial and idiopathic forms of PD in which mitochondrial dysfunction seems to play a major role [38]. Indeed, α-syn can be relocated at the mitochondria where it is able to disrupt mitochondrial bioenergetics and interfere with mitochondrial biogenesis [38]. However, mitochondrial impairment may also be an early event in α-syn nucleation and deposition and potentially favors its pathological aggregation [38]. α-syn is highly expressed in neurons; however, astrocytes process this aberrant protein in a more efficient way [39]. Indeed, the exposure of murine astrocyte and neuron co-cultures to oligomeric α-syn induces co-localization of α-syn oligomers in glial cells and promotes the internalization of larger amounts of this protein in astrocytes [39]. Furthermore, following exposure to oligomeric α-syn, aberrant mitochondrial morphology and enhanced cell death were observed [39]. Of note, astrocytes were still able to survive days after α-syn exposure, likely because these cells rely on glycolysis-dependent metabolism, and neuronal cell demise occurred following astrocytic mitochondrial dysfunction and cytokine release [39]. Upon α-syn uptake, astrocytes triggered the clearance of α-syn oligomers via lysosomal degradation. However, in the setting of incomplete α-syn digestion, accrual of intracellular misfolded protein and mitochondrial impairment ensued [39]. Therefore, when the ability of astrocytes to dispose toxic α-syn species is overwhelmed, the persistence of α-syn deposits induces cellular dysfunction [39]. In keeping with a decline in astrocyte quality as a feature of PD pathophysiology is also the severe mitochondrial impairment observed in astrocytes from PTEN-induced kinase 1 (PINK1) knockout mice [40]. Similarly, astrocytes from Parkin-deficient mice showed aberrant mitochondrial activity and were unable to contribute to neuronal differentiation, thus supporting the hypothesis of a role of glia in PD pathogenesis [41]. Finally, the mitochondrial protein deglycase DJ-1, a sensor of oxidative stress, is crucial for preserving astrocyte mitochondrial homeostasis [42].

Taken as a whole, these findings support a central role of microglia and astrocytes in PD pathophysiology, which is being increasingly appreciated despite a traditional neuro-centric view of the disease. Moreover, the release of mitochondrial-derived damage-associated molecular patterns (DAMPs) as mediators of neurodegeneration is also emerging [43] and will be discussed in more depth in the next section.

## 3. Mitochondrial-Derived Vesicles: Alleviating Cell’s Oxidative Burden

Dysfunctional mitochondria produce greater amounts of ROS, which have detrimental effects on the cell’s physiology by promoting aberrant protein folding and intracellular accrual of toxic protein aggregates (i.e., Aβ_1–42_, α-syn, huntingtin, and Tau) [44]. If not disposed promptly, this waste material impacts cellular activities and can spread to neighboring cells and the extracellular environment [44]. The endo-lysosomal system, which is known for delivering portions of plasma membranes to the endosomal compartment for recycling, has recently been recognized as having a role in the orchestration and execution of autophagy, as a complementary mechanism to guarantee cellular quality control. Mitochondrial homeostasis can also be regulated via this pathway, and its dysfunction has been proposed as a mechanism contributing to age-related conditions, including neurodegeneration [44].

Vesicular transport is a highly conserved type of cell/organismal communication spanning different life kingdoms [45]. Among these, bacteria, which are the best studied, use vesicles to regulate quorum sensing and the exchange of signaling molecules that modulate gene expression and coordinate the behavior of bacterial communities [45]. Bacterial vesicles are used to transport proteins over long distance, promote host invasion, form protective biofilms that allow survival and growth of microorganisms in adverse environmental conditions [46,47].

Owing to their endosymbiotic origin, mitochondria hold multiple bacterial features, including a circular genome, the mitochondrial DNA (mtDNA), embedded into the organelle matrix. The double membrane of mitochondria makes them semi-autonomous from the nucleus also via an independent translation machinery. The ability of generating mitochondrial-derived vesicles (MDVs) of about 70–150 nm in diameter carrying mitochondrial proteins indicates that vesicular transport may be an additional bacterial ancestry trait conserved through evolution [48,49,50,51,52].

Upon formation, MDVs can pursue two different fates: (1) they can deliver their cargo to peroxisomes [48,49,52]; or (2) they shuttle mitochondrial proteins along the endocytic pathway via late endosomes/multivesicular bodies (MVBs). Here, mitochondrial components are directed to lysosomes for degradation [48], thereby suggesting the involvement of MDVs in mitochondrial quality control (MQC) [53,54,55,56,57]. An additional route that can be pursued by MVBs is that of cell surface toward which MVBs are directed to fuse their membranes with the plasma membrane and release MDVs as EVs [54].

MDVs directed to the endocytic pathway have been identified among the vesicle subtypes constitutively produced at a high rate in cardiac myoblasts grown in galactose-containing media [48,50], an experimental setting forcing cells to rely on oxidative metabolism and overusing the mitochondrial protein machinery [48,50]. Under these circumstances, MDV generation was activated only minutes after mitochondrial-induced oxidative stress via antimycin-A and xanthine/xanthine oxidase treatment compared to activation of mitophagy hours/days after stressor exposure [48,50,57]. Moreover, a remarkable budding of MDVs was observed following doxorubicin mitochondrial and cardiac toxicity [50]. These results support a role for MDVs as a first line of defense against oxidative stress and indicate MDV generation as a possible source of biomarkers in conditions of mitochondrial distress [53,54].

Recently, Vasam et al. [58], using budding/reconstitution in vitro assays followed by proteomics analyses, identified protein signatures in MDVs generated by cardiac cells under oxidative stress. These vesicles were enriched in ETC constituents, metabolic enzymes of the Krebs cycle and fatty acid metabolism, autophagy-related mediators, proteins with Fe–S clusters, hyper-reactive cysteine residues, and antioxidant systems [58]. Some of these MDV-associated proteins were also identified within EVs, thus indicating that such molecules may reflect mitochondrial stress and possible biomarkers of mitochondrial damage [58]. The proteomic characterization of MDVs under oxidative stress conditions indicates that selective molecule incorporation most likely relies on the proximity of cargo to ROS-emitting sites [58]. Mitochondria produce ROS at multiple sites, including the flavin NADH:ubiquinone oxidoreductase core subunit V1–3 (NDUFV1–3), the Fe–S NADH:ubiquinone oxidoreductase subunit 1–8 (NDUFS1–8) of complex I [59], the subunits of the ubiquinol-cytochrome c reductase of complex III (UQCRFS1, UQCRC1, UQCRC2) [59,60], and the succinate dehydrogenase complex flavoprotein subunit A of complex II (SDHA) [61]. Furthermore, the mitochondrial glycerol-3-phosphate dehydrogenase (GPDH), the electron transport system of fatty acid oxidation [62], and the dihydrolipoamide dehydrogenase of 2-oxoacid dehydrogenase complexes are also important sites of ROS production [63]. The identification of many of these proteins within MDV cargoes and the observation of their increase following antimycin A treatment indicate that these components may be the most susceptible to damage when high levels of ROS are produced.

More encouraging is the fact that these results are in line with pioneer studies aimed at evaluating the profile of MDVs as markers of mitochondrial dysfunction in people with age-associated conditions characterized by decline in MQC pathways [64,65,66]. In particular, low levels of adenosine triphosphate 5A (ATP5A), NDUFS3, and SDHB have been detected in small EVs isolated from serum of older adults with PD or physical frailty and sarcopenia compared with age-matched controls [64,65,66].

Similar to PD, AD is a neurodegenerative condition featured by the accrual of misfolded proteins and their spreading across neurons and glial cells within the CNS. Among the pathogenic mechanisms of AD, an altered amyloid protein precursor (APP) trafficking involving endosomal vesicular transport is actively investigated. Indeed, the endosomal sorting complexes required for transport (ESCRT) pathway is responsible for directing APP sorting into ILVs [67,68]. The characterization of EVs from neuronal cultures revealed uptake of isolated EVs containing Tau and Aβ oligomers by these cells [69,70] with cytotoxic effects and neuronal spreading [70]. Upon EV reception, target cells acquire the ability to regulate further EV uptake and secretion, thereby boosting the spreading of toxic proteins [71]. Finally, the analysis of neuronal EVs isolated from children with Down syndrome (DS) revealed alterations in insulin-signaling/mTOR pathways [72]. Such changes may represent early events in brain dysfunction associated with DS and likely contribute to cognitive decline in this progeroid condition [72]. Although mitochondrial dysfunction has also been recognized as a major contributor to AD [73], additional work is warranted on the possible role of MDVs in this condition.

The analysis of EV trafficking and the characterization of MDVs from different cell sources in conditions characterized by MQC decline may offer novel pathways for biomarker discovery and therapeutic development.

## 4. Mitochondria, Inflammation, and Astrogliosis

Age-related declines in glial and astrocytic functions have been linked to mitochondrial dysfunction and inflammation. Inflammatory mediators released by activated glial cells can modulate mitochondrial function, thereby establishing a crosstalk between mitochondrial dysfunction and neuroinflammation [43]. In this setting, mitochondrial DAMPs may have the dual role of mediating neurodegeneration and amplifying neuroinflammation [43].

Among the many mitochondrial-derived DAMPs, ROS have been implicated in triggering sterile inflammation in astrocytes via innate immunity. The activation of the NLR Family Pyrin Domain Containing 3 (NLRP3) protein of the inflammasome and the recruitment of caspase-1 complex at the mitochondria are major players in this response [74]. Via caspase-1 activity, the cleavage of IL-1β and IL-18 precursors occurs, and the release of IL-1β and IL-18 is promoted, which can mediate pyroptosis and cell death [75]. However, a role of astrocytes in mediating antigen presentation and T-cell activation has also been proposed [76].

Regardless of the inflammatory mechanism involved, a persistent activation of inflammation through mitochondrial dysfunction has been reported to induce astrocyte hyperactivation, which further bursts inflammation and may aggravate neuronal damage [77]. This condition is called reactive astrogliosis and is a pathologic feature of several CNS disorders.

**Figure 1:**
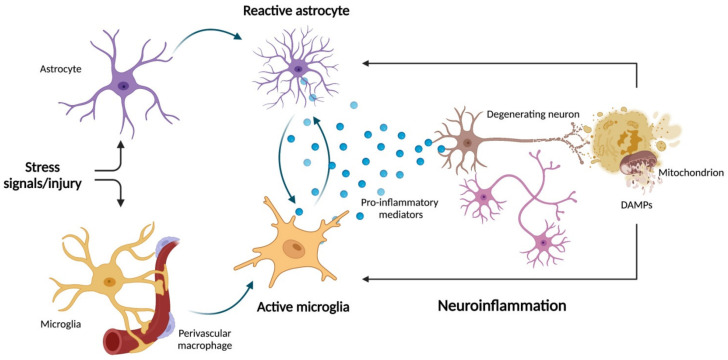
Schematic representation of microglia–astrocyte crosstalk during neuroinflammation and neurodegeneration. Following stress signals and injuries, aged glial cells and astrocytes may trigger astrogliosis. This process has been linked to crosstalk between mitochondrial dysfunction and neuroinflammation. The release of inflammatory mediators by activated glial cells can impinge on mitochondrial function, which in turn can promote further release of neuronal pro-inflammatory damage-associated molecular patterns (DAMPs). In this setting, mitochondrial DAMPs may have the dual role of mediating neurodegeneration and amplifying neuroinflammation. Created with BioRender.com, accessed on 12 May 2022.

Oxidative/nitrosative stress is crucial in mediating astrogliosis via astrocyte-mediated inflammatory response also implicated in neurodegeneration [78]. However, other mitochondrial processes can contribute to inflammation in the setting of astrogliosis. For instance, the uncoupling protein 2 (UCP2) has been reported to regulate astrocyte inflammation via the NLRP3 pathway by modulating levels of mitochondrial ROS [79]. In further support of a mitochondrial-mediated inflammatory astrogliosis is the observation that the release of mtDNA at the cytosolic or extracellular level triggers inflammation via the activation of cyclic GMP—AMP synthase (cGAS)—stimulator of interferon genes (STING) pathway and NLRP3 inflammasome [80]. In this regard, newly synthesized oxidized mtDNA has been reported to bind and activate the NLRP3 complex in the cytosol [81]. In addition, altered protein levels of complex I and depletion of transcription factor A, a histone-like protein for mtDNA, have been associated with α-syn pathology in nigral dopaminergic neurons obtained post mortem from patients with sporadic PD [82]. Moreover, single-nuclei RNA sequencing of midbrain neurons from people with idiopathic PD identified clusters of disease-specific cells and glial activation as pivotal mechanisms involved in PD pathophysiology [83]. However, the causal relationship between mitochondrial dysfunction, α-synucleinopathy, astrogliosis, and neurodegeneration warrants investigation. Preliminary results obtained in astrocytes derived from induced pluripotent stem cells (iPSCs) from healthy people showed that the uptake of high molecular-weight α-syn fibrils conferred a reactive antigen-presenting phenotype to these cells [84]. α-syn exposure of iPSCs also impaired mitochondrial respiration, an effect that was even more pronounced in iPSC-derived astrocytes from PD patients harboring mutations in the mitophagy-related Parkin gene [84]. In a recent study, Joshi et al. [37] showed that the release of dysfunctional and fragmented mitochondria by the microglia was able to trigger neuronal damage and propagate neuronal death via the activation of naïve astrocytes into the pro-inflammatory A1 state [37]. Hence, a crosstalk between microglia and astrocytes rather that a decline in the activity of one of the two cell types alone may be crucial in mediating neuroinflammation and neuronal cell death. Furthermore, the identification of mitochondrial DAMPs as part of innate immunity-driven neuroinflammation may indicate pathways for developing novel therapeutics against neurodegeneration. Indeed, strategies aimed at blunting mitochondrial fragmentation in microglia and therefore inhibiting the release of dysfunctional mitochondria into the brain milieu, without affecting the release of healthy neuroprotective mitochondria, may represent new therapeutic venues (Figure 1).

## 5. Antioxidant Supplementation: A Strategy against Neurodegeneration?

Oxidative stress is a major contributor to brain aging and neurodegeneration. Therefore, supplementation with endogenous or exogenous antioxidants might confer cognitive benefits. Coenzyme Q10 (CoQ10), glutathione (GSH), melatonin, vitamins, polyunsaturated fatty acids (PUFAs), polyphenols, and mitoquinone Q (MitoQ) are among the molecules that have been investigated for the neuroprotective potential and are discussed in the following subsections.

### 5.1. Coenzyme Q10

CoQ10 is a benzoquinone ring holding a side chain of 10 isoprene units synthetized endogenously. CoQ10 is produced at higher rates in tissues characterized by sustained metabolic activity and high energy demands [85]. This antioxidant compound has been reported to exert its neuroprotective effects by increasing mitochondrial function and promoting lipid reduction, thus protecting the body against the build-up of fats due to unhealthy diet habits [86,87,88]. CoQ10 has also been attributed anti-inflammatory properties through the inhibition of nuclear factor κB (NF-κB) and the release of pro-inflammatory cytokines by endothelial cells of the blood–brain barrier [89]. Moreover, CoQ10 reduces the expression of genes related to endoplasmic reticulum (ER) stress (e.g., calreticulin) and altered ER–mitochondria communication [90].

### 5.2. Glutathione

GSH is a well-characterized endogenous antioxidant. This compound is a glycine–glutamine–cysteine peptide with a thiol group that allows preserving the cellular redox state through detoxification reactions. GSH is abundant in microglia and has a key role in the expression and regulation of many antioxidant enzymes [91]. A regulated synthesis of GSH in astrocytes is also crucial to replenish the neuronal GSH pool via astrocyte–neuron crosstalk. Studies have shown that a reduction in GSH levels, GSH-S-transferase (GST), superoxide dismutase (SOD) activity, and GSH/oxidized-GSH ratio characterizes brains of patients with AD [92]. GSH limits Aβ-induced mitochondrial membrane depolarization in human cortical neuronal HCN-1A cells [93] and acts as a mitochondrial aconitase activator [94]. Lower levels of GSH and higher oxidative stress have been found in patients with PD [95]. GSH supplementation was found to have a positive effect on the disruption of α-syn aggregates in the brain of a transgenic mouse model overexpressing human α-syn bearing the A53T mutation (prnp.aSyn.A53T) [96]. Finally, co-treatment with γ-glutamylcysteine (GGC), a precursor of GSH, and Aβ_40_ oligomers of astrocyte cultures increased SOD and GSH peroxidase activity, as well as total cellular antioxidant capacity [97]. GGC supplementation was also shown to increase the levels of anti-inflammatory cytokines and reduce metalloproteinase activity in astrocytes treated with oligomeric Aβ_40_ [97].

### 5.3. Melatonin

Another endogenous compound holding antioxidant activity is melatonin, a key hormone in the regulation of circadian rhythm. Melatonin seems to have a protective action in different animal models by maintaining mitochondrial membrane potential, increasing antioxidant enzymatic (e.g., SOD, catalase) and non-enzymatic defenses (i.e., GSH), inhibiting ROS overproduction, increasing ATP production, decreasing calcium concentrations, and enhancing mitochondrial complex I activity [98,99,100,101,102].

### 5.4. Vitamins

Microglia-mediated response to oxidative stress may become insufficient, and supplementation with natural compounds that improve cellular antioxidant defense may be beneficial. Several compounds extracted from fruits, vegetables, and fish have been attributed a protective role against oxidative damage.

Vitamin E is abundant in sunflower, safflower, and soybean oil, sunflower seeds, almonds, peanuts, beet greens, collard greens, spinach, pumpkin, asparagus, mango, and avocado. Vitamin E preserves biological membranes from oxidation and modulates enzymes that reduce ROS/RNS build-ups [90]. Vitamin E is a family of eight natural forms that include four tocopherols and four tocotrienols divided into α, β, γ, and δ forms. The hydroxyl group of the aromatic ring of vitamin E neutralizes radicals or reactive species by ceasing a hydrogen atom [90]. The administration of α-tocopherol was shown to reverse the altered synaptic plasticity observed in PD mice [103]. Moreover, a recent study indicated that α- and β-tocopherol, δ-tocotrienol, total tocopherols, total tocotrienols, and total vitamin E may be involved in the pathogenesis of AD [104].

The precursor of vitamin A, the β-carotene, is found in yellow, orange, and green leafy fruits and vegetables (e.g., carrots, spinach, lettuce, tomatoes, sweet potatoes, broccoli, cantaloupe, pumpkin) and has positive effects against oxidative stress and neurodegeneration [105,106]. Indeed, low β-carotene plasma concentrations have been found in people with AD [107] and PD [108] compared with healthy controls. These findings have recently been confirmed by an in vitro study demonstrating that β-carotene reduces oxidative stress and pro-inflammatory cytokines in mononuclear cells of people with AD [109]. However, proper timing of vitamin E supplementation in relation to the time course of AD pathology should also be considered. Indeed, an association between higher α- and γ- tocopherol levels and lower total and activated microglia density has been identified in the human cortex, suggesting a microglia-mediated beneficial effect on the slowly accumulating AD neuropathology [110]. However, improvements in microglial activation should better be obtained in the early stages of AD, as microglia is crucial for clearing soluble Aβ and creating protective barriers around Aβ plaques [111,112]. In later AD stages, instead, persistent microglial activation can reinforce tau pathology and have negative effects on neurons and synapses [110,113].

Finally, many fruits and vegetables are rich in vitamin C (ascorbic acid). Vitamin-C-rich fruits include citrus fruits, such as oranges, grapefruit, and lemon, kiwi, blackcurrants, strawberries, and guava. As for vegetables, broccoli, cauliflower, cabbage, cooked kale, Brussels sprouts, and Chinese cabbage are good sources of this vitamin. Vitamin C acts as a scavenger of free radicals produced as by-products of cell metabolism and down-regulates the activity of the nicotinamide adenine dinucleotide phosphate (NADPH) oxidase, thereby reducing ROS production [114]. In addition, vitamin C contributes to the maintenance of both mitochondrial integrity and function by preventing abnormal mitochondria morphology [93,115]. Interestingly, supplementation with vitamin C has been reported to mitigate the degeneration of dopaminergic neurons and locomotor deficits in an animal model of PD [116].

### 5.5. Polyunsaturated Fatty Acids

Marine-based fish and fish oil are the most important sources of n-3 PUFAs (e.g., docosahexaenoic acid (DHA), eicosapentaenoic acid (EPA)), with a well-known role in neuronal development and growth, as well as in mitochondrial biogenesis and the regulation of genes involved in brain oxidative metabolism [90]. A greater intake of DHA has been associated with partial recovery of the dopaminergic system, suggesting both neuroprotective and neurorestorative capacity in PD patients [90]. In fact, DHA can reduce neuroinflammation, mitochondrial dysfunction, and oxidative stress caused by α-syn alterations [117,118,119]. Preclinical studies in AD models have shown an improvement in mitochondrial function following PUFA treatment, with positive effects on ROS production, cytochrome c release, and caspase-3 activation [120]. A recent study also indicated a favorable effect of a multi-nutrient intervention containing both DHA and EPA in slowing cognitive decline, brain dysfunction and atrophy, and disease progression in AD [121].

### 5.6. Polyphenols

Fruits, vegetables, cereals, spices, olive oil, and wine are rich in polyphenols, a class of compounds characterized by an aromatic ring with at least one hydroxyl group able to chelate divalent metals (e.g., copper, zinc, and iron) [122]. These compounds can attenuate mitochondrial dysfunction through the regulation of calcium homeostasis, preservation of the membrane potential, and promotion of cytochrome c release into the cytosol during apoptosis [123].

Curcumin is a polyphenol compound derived from the turmeric plant (*Curcuma longa* L.) and has a protective role in astrocytes, neurons, microglia, and different regions of the CNS, such as hippocampus, mesencephalon, cerebral cortex, and spinal cord [124,125,126], by preventing the production of hydrogen peroxide and nitric oxides [127]. Interestingly, curcumin acts as mitochondrial antiapoptotic agent through the inhibition of caspase-3 and caspase-9 activities and cytochrome c release, and protects mitochondrial integrity and function via reduction in ROS production through amelioration of complex I activity [128,129]. Among synthetic derivative compounds from curcumin, CNB-001 seems to prevent mitochondrial damage induced by rotenone in human neuroblastoma SK-N-SH cells by inhibiting the mitochondrial apoptotic pathway and maintaining mitochondrial structure [130,131]. Moreover, glutamoyl diester of curcumin has been shown to preserve mitochondrial membrane potential and to inhibit ROS production in brain mitochondria of mice with peroxynitrite-induced PD [132].

Resveratrol is a natural polyphenol found in grapes, berries, peanuts and, above all, red wine. Resveratrol modulates mitochondrial bioenergetics in primary fibroblasts cultures from PD patients with parkin mutations (PARK2) by increasing complex I and citrate synthase activity, basal oxygen consumption and ATP production, and reducing lactate content [133]. With regard to AD, resveratrol treatment seems to normalize mitochondrial amount and decrease the abnormal expression of peroxiredoxins and mitochondrial structural genes in Aβ_25–35_-induced N2a mouse cells [134].

Curcumin and resveratrol bear the weakness of a limited bioavailability. After oral intake, these compounds are rapidly metabolized in the liver and intestine and are promptly disposed by the body. Due to their low bioavailability, curcumin and resveratrol barely cross the blood–brain barrier, which impacts their neuroprotective potential [90]. New strategies have been developed to improve the bioavailability of these polyphenols, such as the use of nanoparticles that can easily reach the blood–brain barrier endothelial cells [135,136,137].

Fruits from Ericaceae are also rich in polyphenols with strong antioxidant properties. Experimental evidence showed that the intake of blueberries, cranberries, and bearberries had a protective effect on the CNS [138]. In particular, an in vitro study reported that blueberries prevented mitochondrial damage associated with Aβ and reduced the accumulation and aggregation of Aβ through NF-κB regulation [139]. In vivo study confirmed the positive effect of cranberries consumption in improving motor coordination and memory in old rats [140].

Several polyphenolic compounds, including flavanols, flavandiols, flavonoids, and phenolic acid, are also found in green tea leaves. In human neuroblastoma SH-SY5Y cell model of 6-hydroxydopamine-induced PD, treatment with green tea polyphenols inhibited the intrinsic apoptotic pathway, reduced ROS production, and ameliorated intracellular calcium concentrations [141]. Finally, treatment with EGb761, an extract from Gingko biloba leaves, improved cognitive performance in people with AD [142].

### 5.7. Mitoquinone Q

Mitochondrial-targeted antioxidants are a relatively new field of research with promising clinical applications. MitoQ, which is obtained by conjugating the lipophilic triphenylphosphonium cation to coenzyme Q [143], offers the advantage of diffusing through the mitochondrial membranes, thereby accumulating within the organelle [144]. MitoQ was effective in preventing loss of spatial memory and delaying early neuropathology in a triple transgenic mouse model of AD, by preserving mitochondrial membrane potential and reducing apoptosis in cortical neurons [145]. In another animal model of AD, MitoQ attenuated cardiolipin depletion and increased ETC function [146]. Moreover, treatment with MitoQ inhibited the mitochondrial apoptotic pathway in human neuroblastoma SH-SY5Y cell model of 6-hydroxydopamine-induced PD [147,148]. Interestingly, treatment with MitoQ increased the activity of several antioxidant enzymes and attenuated neurological deficits in a mouse model of traumatic brain injury [149].

Although preclinical studies reported promising effects of antioxidant supplementation against neurodegeneration, results from clinical trials using vitamins (vitamin C and E) and CoQ10 in people with PD or AD yielded conflicting results [150,151,152]. Results from these trials indicate that the clinical benefits of antioxidant supplementation may be marginal and likely more evident in people with mild to moderate disease. Further studies are warranted to explore whether specific combinations of supplements given early during the disease course may produce meaningful clinical benefits by targeting multiple processes (i.e., the crosstalk between microglia and astrocytes) rather than a single pathway.

## 6. Conclusions

The knowledge of the pathophysiological mechanisms associated with neurodegeneration may help develop pharmacological and nutraceutical interventions to counteract cognitive decline. The decline in processes involved in preserving the cell’s quality is a critical event upstream of the accumulation of oxidative damage and proteotoxic aggregates that may burst astrocytes-mediated secretion of pro-inflammatory cytokines and lead to neuroinflammation. In this regard, the analysis of mitochondrial dysfunction and, in particular, MDV trafficking, warrant further investigation. Targeting the crosstalk between microglia and astrocytes has emerged as a novel promising tool to modulate oxidative damage, a relevant pathophysiological mechanism involved in neurodegeneration. In fact, neuroglia can act as a major source of ROS, causing oxidative damage and mediating secondary damage, such as neuroinflammation, excitotoxicity, and blood–brain barrier disruption. On the other hand, ROS affect the phenotype of glial cells by activating astrocytes and promoting polarization of microglia, making interventions to modulate ROS/RNS production a promising, albeit challenging, strategy against neurodegeneration.

## Data Availability

No data were generated for the present study.
